# Safety and efficacy of toll-like receptor agonists as therapeutic agents and vaccine adjuvants for infectious diseases in animals: a systematic review

**DOI:** 10.3389/fvets.2024.1428713

**Published:** 2024-09-17

**Authors:** Harriet Oboge, Victor Riitho, Mutono Nyamai, George P. Omondi, Anna Lacasta, Naftaly Githaka, Vishvanath Nene, Gabriel Aboge, S. M. Thumbi

**Affiliations:** ^1^Department of Public Health Pharmacology and Toxicology, Faculty of Veterinary Medicine, University of Nairobi, Nairobi, Kenya; ^2^Centre for Epidemiological Modelling and Analysis, University of Nairobi, Nairobi, Kenya; ^3^Paul G. Allen School for Global Health, Washington State University, Pullman, WA, United States; ^4^Animal and Human Health, International Livestock Research Institute, Nairobi, Kenya; ^5^Feed the Future Innovation Lab for Animal Health, Washington State University, Pullman, WA, United States; ^6^Institute of Tropical and Infectious Diseases, University of Nairobi, Nairobi, Kenya; ^7^Department of Clinical Studies, Faculty of Veterinary Medicine, University of Nairobi, Nairobi, Kenya; ^8^Institute of Immunology and Infection Research, School of Biological Sciences, University of Edinburgh, Edinburgh, United Kingdom

**Keywords:** toll-like receptor agonists, efficacy, safety, therapeutics, adjuvants, vaccines, infectious diseases, animals

## Abstract

**Introduction:**

Strengthening global health security relies on adequate protection against infectious diseases through vaccination and treatment. Toll-like receptor (TLR) agonists exhibit properties that can enhance immune responses, making them potential therapeutic agents or vaccine adjuvants.

**Methods:**

We conducted an extensive systematic review to assess the efficacy of TLR agonists as therapeutic agents or vaccine adjuvants for infectious diseases and their safety profile in animals, excluding rodents and cold-blooded animals. We collected qualitative and available quantitative data on the efficacy and safety outcomes of TLR agonists and employed descriptive analysis to summarize the outcomes.

**Results:**

Among 653 screened studies, 51 met the inclusion criteria. In this review, 82% (42/51) of the studies used TLR agonists as adjuvants, while 18% (9/51) applied TLR agonist as therapeutic agents. The predominant TLR agonists utilized in animals against infectious diseases was CpG ODN, acting as a TLR9 agonist in mammals, and TLR21 agonists in chickens. In 90% (46/51) of the studies, TLR agonists were found effective in stimulating specific and robust humoral and cellular immune responses, thereby enhancing the efficacy of vaccines or therapeutics against infectious diseases in animals. Safety outcomes were assessed in 8% (4/51) of the studies, with one reporting adverse effects.

**Discussion:**

Although TLR agonists are efficacious in enhancing immune responses and the protective efficacy of vaccines or therapeutic agents against infectious diseases in animals, a thorough evaluation of their safety is imperative to in-form future clinical applications in animal studies.

**Systematic review registration:**

https://www.crd.york.ac.uk/prospero/display_record.php?RecordID=323122.

## 1 Introduction

Vaccination is currently the most effective strategy for controlling infectious diseases amidst the rising global concerns over the increasing risk of antimicrobial resistance, especially in low- and middle-income countries with poor pharmaceutical regulatory frameworks (1, 2). On the other hand, there are emerging public health concerns arising from the emergence of new pathogen strains and the re-emergence of many infectious diseases (3). Designing effective vaccines for infectious diseases, especially for emerging pathogens remains challenging. To improve the immune potency of existing vaccines, there is a need to understand host-pathogen immune responses and develop novel vaccines based on this knowledge (4, 5).

Traditionally, vaccines have been developed as live attenuated, live whole organisms, killed, and inactivated toxoids from organisms (6). Live attenuated or live whole cells vaccines, despite concerns over incomplete attenuation and associated risks of disease after vaccination, as in the cases of yellow fever and measles, contain sufficient PAMPs thus inducing adequate immunostimulatory activity (6–8). For example, for *Theileria parva*, an intracellular pathogen, a live vaccine, the Muguga cocktail vaccine containing three *Theileria parva* stocks has fairly good protection, albeit accompanied by creating a carrier state in vaccinated cattle and potential field parasite diversity changes and potential for disease introduction in previously naïve populations (9, 10). On the other hand, subunit vaccines containing protein or glycoprotein pieces of a pathogen, despite offering improved safety and prospects for quick development of new vaccines are comparatively poor immunogens (11, 12). Improved immunogenicity is achieved by the addition of adjuvants (13, 14). However, the use of adjuvants maybe accompanied by adverse effects. For instance, water-in-oil immersion adjuvants such as Freund’s complete adjuvant (FCA) and Freund’s incomplete adjuvant (FIA), despite their high potency as immune stimulants, induce severe adverse effects, including abscess formation, granulomas, inflammation at the site of injection, severe pain, and fever (15). On the other hand, aluminum salts, despite their immunostimulatory boosting activities, weakly induce Th1 immunity, a critical response for intracellular parasites such as *Theileria* spp. and *Leishmania* spp., among other pathogens (16, 17). Thus, finding an immune-potent adjuvant with minimal or no side effects is essential.

Presently, there exists a heightened understanding of the dynamics in host-pathogen interactions, resulting in the identification of alternative adjuvant formulations and therapeutics against various microbes (6). The discovery of Pathogen Associated Molecular Patterns (PAMPs) and their role in immunomodulation has been a ripe area for research (18, 19). PAMPs are conserved highly expressed functional microbial components, recognized by receptors in humans and animals (20). In responding to infections, the receptors that bind to PAMPs, known as Pattern Recognition Receptors (PRRs), recognize these conserved microbial components and initiate immune cascades, producing proinflammatory and antimicrobial responses and chemotactic factors (21). Toll-like receptors (TLRs), a type PRRs, are located on both the cell surface and within the endosomes. They identify PAMPs, including carbohydrates, nucleic acids, lipids, and proteins (20). The TLRs are expressed on and in cells of the innate immune system of mammals, such as dendritic cells, macrophages, monocytes, and cells of the adaptive immune system such as B cells (22). TLRs found on the cell surfaces recognize surface-associated PAMPs, while those in the endosomes detect nucleic acids. There are 10 TLRs (TLR 1 to TLR 10) in mammals, with distinct functions in the innate immune system, apart from mice, which have 13 (23). In the avian species, chicken TLRs are the most studied, having slightly different TLRs from mammals and mice, including TLR1La, TLR1Lb, TLR2a, TLR2b, TLR3, TLR4, TLR5, TLR7, TLR21, and TLR15 (24). TLRs detect a variety of PAMPs: TLR1/TLR2 binds peptidoglycans from Gram-positive bacteria; TLR2/TLR6 binds to diacylated lipopeptides; TLR3 binds to dsRNA, TLR4 binds to lipopolysaccharide on Gram-negative bacteria outer membrane; TLR5 binds to flagellin of motile bacteria; TLR7/8 binds to ssRNA; TLR9 recognizes unmethylated juxtaposed cytosine and guanine nucleotides (CpG) of bacterial DNA (25, 26). The binding of TLR ligands on antigen-presenting cells (APCs) promotes innate inflammatory responses that induce adaptive immunity, rendering TLRs ideal targets for developing effective therapeutic agents and vaccine adjuvants (24).

With the growing evidence of the active involvement of TLRs in the immune response of animals to infection, TLR agonists gained significant interest in development of vaccines and therapeutics in animals (27). Activation of the immune system by some TLR agonists has been documented to lead to detrimental side effects linked to unintended expansion of the adaptive immune cells, resulting in susceptibility to an infection or reduced efficacy of vaccines (28). It is crucial, therefore, to comprehensively underscore the adverse effects of TLR agonists, a vital consideration in their selection as therapeutics or vaccine adjuvants (28, 29). Thus, we conducted a systematic review to assess the efficacy and safety of TLR agonists as therapeutic agents and vaccine adjuvants for infectious diseases in animals to answer the following questions: (i) are TLR agonists efficacious therapeutic agents or vaccine adjuvants for infectious diseases in animals? (ii) Are TLR agonists safe for animal use as therapeutic agents and vaccine adjuvants?

## 2 Methodology

This systematic review used the Preferred Reporting Items for Systematic Reviews and Meta-Analysis (PRISMA) guidelines. The review protocol is registered at the International Prospective Register of Systematic Reviews (PROSPERO); protocol registration number CRD42023323122 (30).

### 2.1 Database sources and search strategy

A systematic search was conducted on January 25, 2023, and followed by an additional search on April 29, 2024 to ensure the search was current and up-to-date. The aim was to identify all the potentially relevant peer-reviewed articles from major electronic databases including PubMed, Embase, and Google Scholar. The systematic search was based on PICO (Population, Intervention, Control, and Outcome) framework and medical subject headings (MeSH) to identify keywords and index terms in which the Boolean operators (“AND,” “OR,” and “NOT”) were utilized to connect the keywords. The keywords used for the general search include: “(tlr or toll-like receptor) and (ligand or agonist) and (vaccine or therapeutic or prophylactic) and (efficacy or safety) and (infection or infectious agent or infectious disease) and (animal or livestock or veterinary) NOT (human or mice or mouse).” The terms for the measures of outcome, efficacy and safety were not included in the search to prevent limitation in the database searches (31). A general search was conducted directly from PubMed and Embase databases, and using Publish or Perish software for the Google Scholar database. Reference lists were retrieved and saved in Microsoft Excel spreadsheets in Comma Separated Value format (CSV) (Table 1).

**Table 1:** Key elements of the systematic search based on the PICO framework.

PICO framework	Research question elements
Population	Animals
Intervention	Toll-like receptor agonists investigated as therapeutic agents or vaccine adjuvants
Comparison	Placebo, alternative treatments or adjuvants, nonvaccinated or nontreated
Outcome	Efficacy of TLR agonists (demonstrated as elicitation of strong humoral and cellular responses that are pathogen specific or specific out come in vaccine or therapeutic efficacy attributed to TLR agonists) and safety profile of TLR agonists (demonstrated as presence or absence of adverse reaction of animals to TLR agonists administration)

### 2.2 Eligibility criteria

The published studies were screened by titles, abstracts, and full-text reviews to determine their eligibility. The studies included in this review must have been peer-reviewed and published, animal studies investigating TLR agonists as a therapeutic agent or vaccine adjuvant, investigating infectious agents or diseases, and conducted at any year, and in any part of the world. Studies were excluded if they were not peer-reviewed and published, involved either human participants or rodents, *ex vivo* and *in vitro* studies or involved *in vivo* studies, studies involving investigation of non-infectious agents or diseases, and studies that lacked full-text availability. Rodent studies were excluded from this review due to significant anatomical, pharmacological, and pathophysiological differences from larger animals, which would limit the translational applicability of their outcomes (32). Additionally, *ex vivo*, *in vitro*, and *in vivo* studies were excluded because of the complexities of correlating their outcomes to those observed in animals (33).

### 2.3 Selection of studies

Initially, duplicates were removed, and the screening questions were developed according to the inclusion and exclusion criteria (as described above). The screening was conducted HO and VR on the Rayyan QCRI platform: Initially an assessment of titles and abstracts and selection of eligible articles using the inclusion and exclusion criteria questions was conducted by HO and VR and subsequently, a full-text review of the selected articles to scrutinize and assess the studies’ eligibility according to the inclusion and exclusion criteria. Any conflicts on the eligibility of any article were resolved by an independent reviewer (AL).

### 2.4 Data extraction, analysis, and presentation

A data collection tool was prepared on Microsoft Excel Version 16.77 (23091003) and relevant data was extracted from the selected articles and cross-checked by two reviewers (HO and VR). Any disagreement by the reviewers on the relevance of the collected data was resolved by involving a third party (AL) to reach a consensus. The data variables extracted are shown in Table 2. Data on study characteristics, efficacy, and safety of Toll-like receptor (TLR) agonists were analyzed qualitatively using R statistical software version 4.1.2 (2021-11-01) and Microsoft Excel version 16.77 (23091003) and summarized using tables and figures.

**Table 2:** Data variables collected from full-text review.

No.	Variables	Description
1	Author	Lead author of the study
2	Year	The year of publication
3	Title	The full title of the study
4	Location	Country where the study was conducted
5	Species	The type of animal model used
6	Disease	The diseases that TLR agonist use was applied
7	Vaccine/treatment	Type of vaccine or treatment administered
8	Toll-like receptor agonist	The type of TLR agonist being investigated
9	Toll-like receptor	Toll-like receptor targeted by the TLR agonist
10	Comparator	Control groups; placebo, active treatment, or no-treatment
11	Route of administration	The route of administration of TLR agonist
12	Type of intervention	Application of TLR agonist as either as adjuvant or therapeutic agent
13	Animal model	Type of animal model used in the studies
14	Study design	The sequence and structure of the experiment
15	Study objective	Assessment of efficacy, safety, and immunogenicity of TLR agonist
16	Sample size	The number of animals included in the studies
17	Animal groups	Number of animal groups used
18	Outcome measures	Efficacy outcome measures; elicitation and enhancement of humoral and cellular responses, safety outcome measures (adverse effects)
19	Summary findings	Summary of key findings

### 2.5 Risk of bias assessment

A Risk of Bias (RoB) assessment for all the studies included in the review was conducted by HO and VR. The Systematic Review Centre for Laboratory Animal Experimentation (SYRCLE) risk of bias (RoB) tool was used to assess the risk of bias across 10 domains (34): sequence generation, baseline characteristics, allocation concealment, random housing, performance blinding, random outcome assessment, detection blinding, incomplete outcome data, selective outcome reporting, and other bias sources. The overall bias was assessed using screening questions and judgment, described in detail elsewhere (34). Reviewer conflicts on identifying the source of risk were resolved by an independent reviewer (AL).

## 3 Results

### 3.1 Study selection

**Figure 1:**
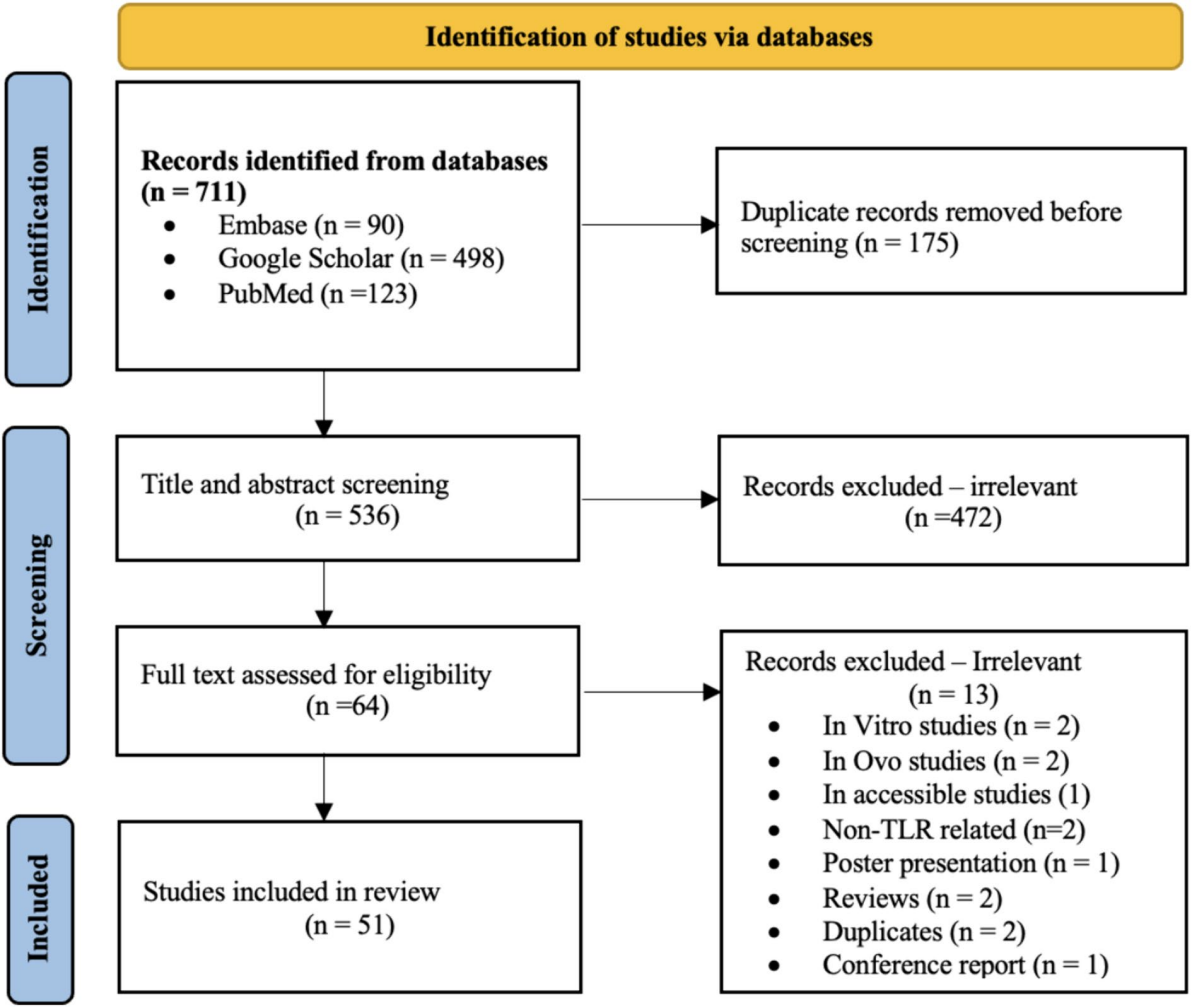
Flow diagram showing the literature search and study selection.

A total of 711 potentially relevant studies were identified in our initial database search. Once duplicates were removed, titles and abstracts from 536 articles were screened, out of which 472 studies that did not meet the inclusion criteria were excluded based on their titles and abstracts. The full-text review was conducted on 64 studies; ultimately, 51 studies met the inclusion criteria and thus were included in this review (Figure 1).

### 3.2 Study characteristics

**Figure 2:**
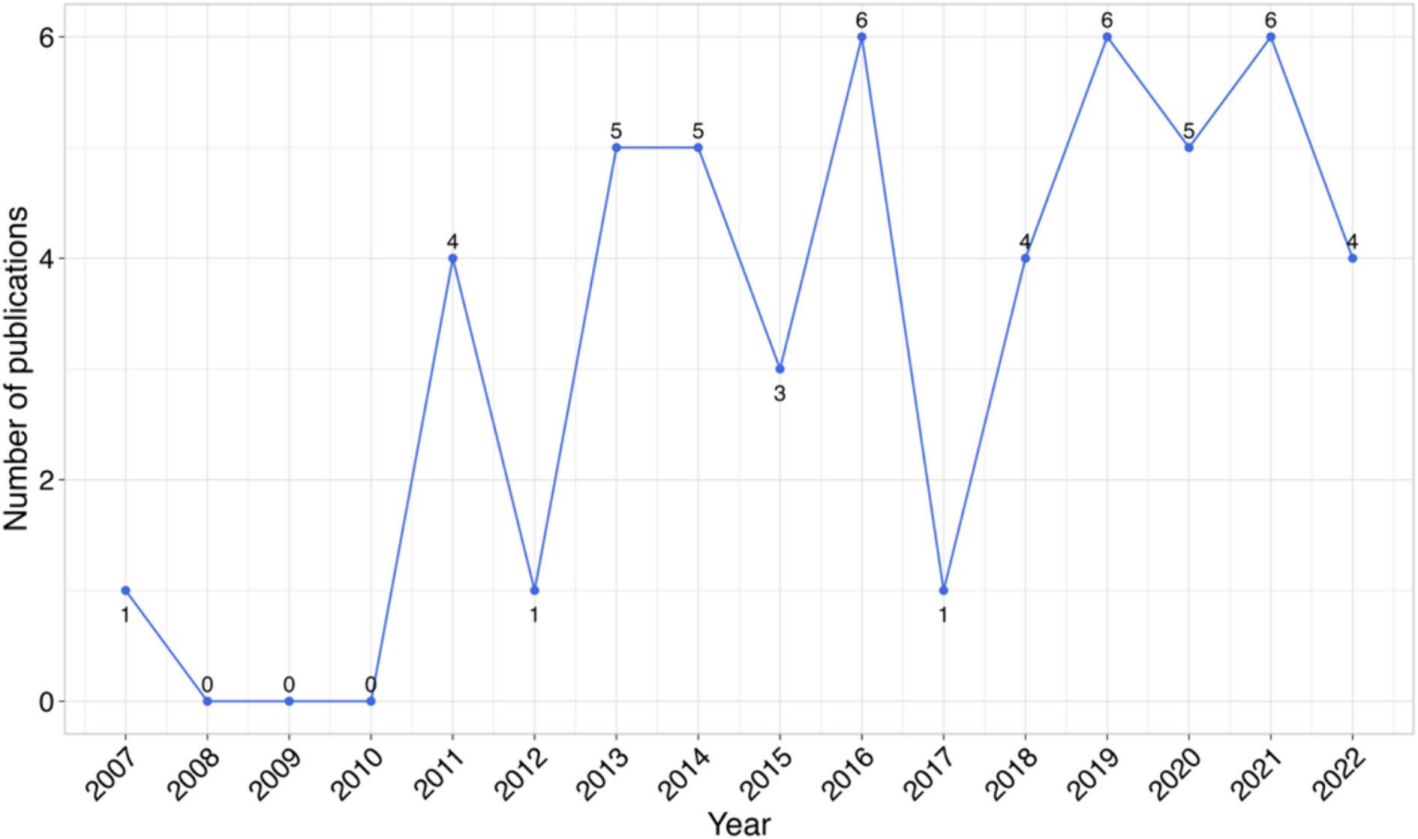
Studies published annually on use of TLR agonists as therapeutic agents and vaccine adjuvants for infectious diseases in animals.

This review included studies published without limitation on the publication year, spanning from 2007 to April 2024. The years 2016, 2019, and 2021 marked the highest number of publications, of six publications, in the respective years (Figure 2). The average age of the publications from the year of publication of the studies to the time we conducted this review was approximately 6 years.

**Figure 3:**
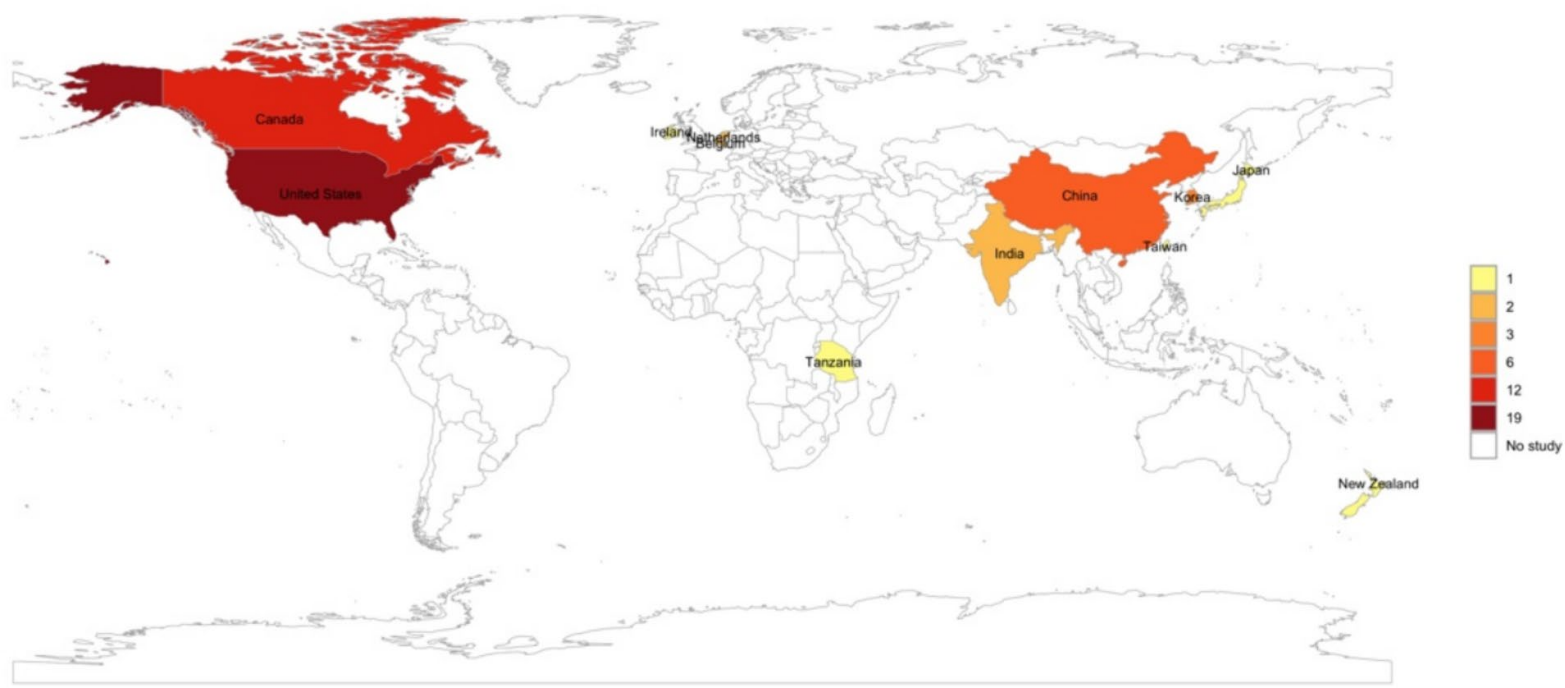
World map created using R statistical software showing the geographical distribution of studies included in the review.

Considering that there was no geographical limitation in the selection of the studies, most of the TLR agonist animal studies for infectious diseases were conducted in the United States (37%) and Canada (26%). Nonetheless, we did not identify any studies conducted in countries across South America, with only one study conducted in Africa, specifically in Tanzania (Figure 3).

Eighty-two percent (42 out of 51) of the studies employed TLR agonists as vaccine adjuvants, while 18% (9 out of 51) utilized TLR agonists for therapeutic purposes. The diseases addressed in the reviewed studies encompassed viral diseases (76%), bacterial diseases (12%), protozoal diseases (6%), and parasitic infections (6%). Inactivated vaccines (41%) and subunit vaccines (33%) emerged as the most frequently utilized types in our review. The studies employed four types of animals, including primates, cattle, swine, and chickens, with non-primates (41%) being the most commonly used animal across the studies reviewed (Table 3). Across the studies, TLR agonists were administered through various routes, such as intra-air sac, intradermal, intramuscular, intranasal, intra-tracheal, oral, and subcutaneous. Given that some studies evaluated the administration of TLR agonists through multiple routes, notably, the intramuscular route (50%) was the most common method of TLR agonist delivery (Table 3).

**Table 3:** Characteristics of studies reviewed.

Variable	TLR as adjuvant *n* (%) *n* = 42	TLR as therapeutic agent *n* (%) *n* = 9	Total *n* (%) *n* = 51
Diseases			
Viral diseases	30 (71)	9 (100)	39 (76)
Bacterial diseases	6 (15)	0 (0)	6 (12)
Protozoal diseases	3 (7)	0 (0)	3 (6)
Parasitic	3 (7)	0 (0)	3 (6)
Type of vaccines/therapeutic			
Inactivated vaccines	17 (41)	0 (0)	17 (33)
Subunit vaccines	14 (33)	2 (22)	16 (31)
Live attenuated vaccines	7 (17)	0 (0)	7 (14)
DNA vaccines	3 (7)	0 (0)	3 (6)
Viral infection therapeutic	0 (0)	7 (78)	7 (14)
mRNA vaccines	1 (2)	0 (0)	1 (2)
Animal models			
Non-human primates	15 (36)	6 (67)	21 (41)
Chicken	16 (38)	2 (22)	18 (35)
Swine	9 (21)	1 (11)	10 (20)
Cattle	2 (5)	0 (0)	2 (4)
Route of TLR administration			
Intramuscular	21 (46)	3 (30)	24 (43)
Subcutaneous	12 (26)	2 (20)	14 (25)
Oral	4 (9)	3 (30)	7 (13)
Intranasal	4 (9)	1 (10)	5 (9)
Intradermal	4 (9)	0 (0)	4 (7)
Intra-air sac	0 (0)	1 (10)	1 (2)
Intratracheal	1 (2)	0 (0)	1 (2)

### 3.3 Application of TLR agonists as adjuvants and drugs

Several TLR agonist were evaluated, including TLR1/2, TLR2, TLR3, TLR4, TLR 2/4, TLR5, TLR7, TLR7/8, and TLR9 agonist. Among these, CpG-ODN, a TLR9 agonist in mammals and a TLR21 agonist in chickens emerged as the predominantly used in 37% (19/51) of the studies (Table 4).

**Table 4:** Toll-like receptor agonist used in the studies reviewed.

TLR agonist	TLR receptors	Number of studies	References
CpG ODN	TLR 9 and TLR 21	19	(38, 63–79)
Resiquimod, 3 M052, 3 M-003, CL097M-012	TLR 7/8	11	(38, 63, 65, 67, 77–83)
LPS, GLA, HSPX, MPLA	TLR 4	11	(35, 36, 65, 71, 74, 84–88)
Poly (I: C)	TLR 3	11	(65, 71, 74, 83, 88–93)
Vesatolimod, Imiquimod, GS-986, SZU10, GS-9620 Adilipoline	TLR 7	10	(37, 83, 84, 87, 94–99)
Flagellin	TLR 5	6	(81, 100–104)
Pam3Cys	TLR 1/2	7	(70, 77–79, 89, 105, 106)
Adilipoline, HSP70c	TLR 2	4	(65, 89, 99, 107)
*Bacillus subtilis* spores	TLR 2/4	1	(108)

### 3.4 Description of efficacy outcomes

**Figure 4:**
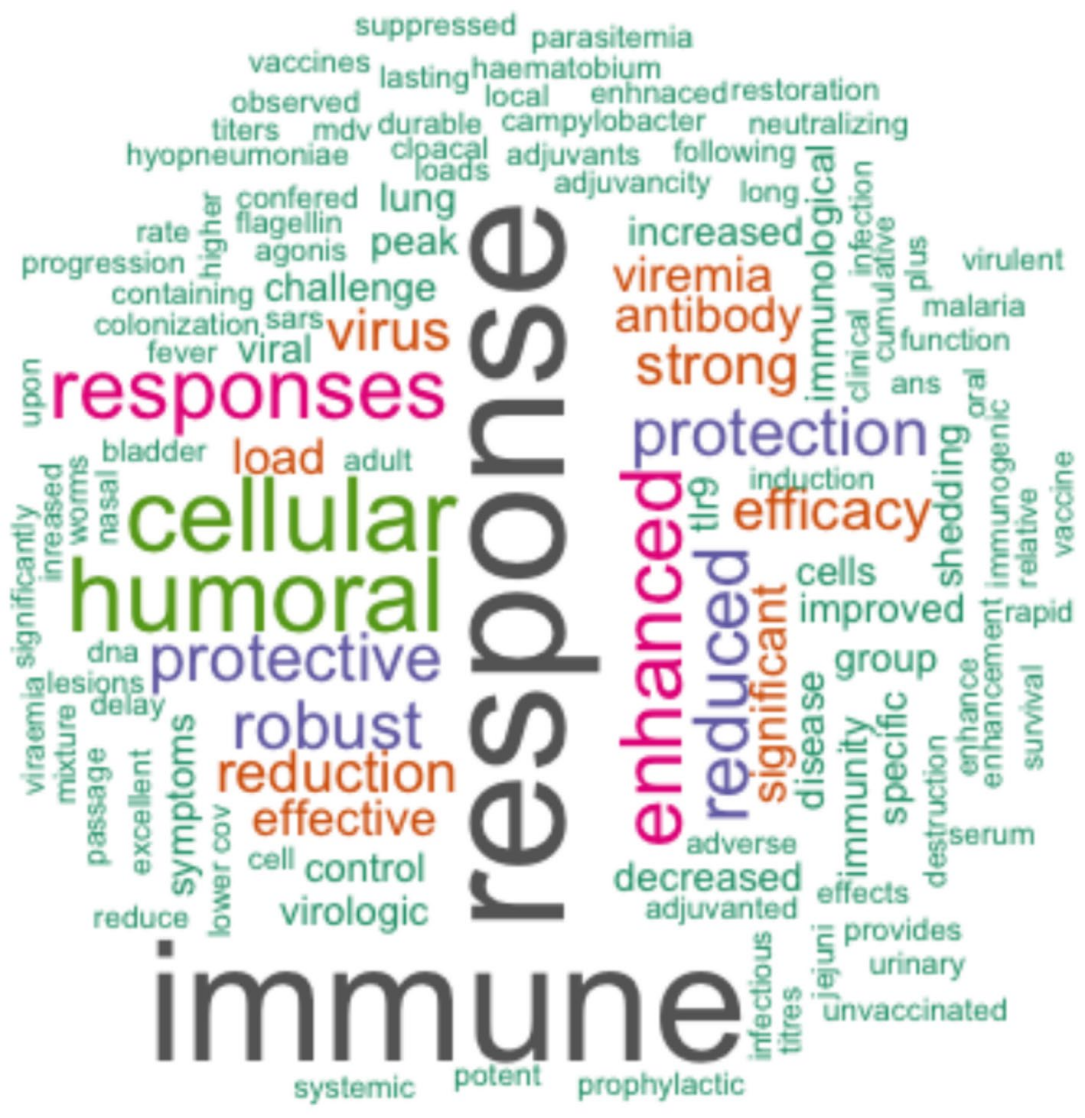
Word cloud created using R statistical software showing a summary of specific outcomes from the reviewed studies on TLR agonists. The size of each word corresponds to the frequency of occurrence of the respective outcome across the studies that had the specified outcome.

Of the studies reviewed, 92% (47/51) assessed the efficacy and immunogenicity of TLR agonists, whereas 8% (4/51) assessed the safety and immunogenicity of TLR agonists. The outcomes of the application of TLR agonists in the studies reviewed were measured by assessment of humoral and cellular responses. 90% (46/51) of the studies reported enhanced antigen-specific humoral and cellular immune responses using TLR agonists compared to control groups. Within the control groups, 45% (23/51) received placebo, 22% (11/51) were administered either antigen without adjuvant, 16% (8/51) were left unvaccinated, while 12% (6/51) received adjuvant without antigen or were administered as a drug, and 4% (2/51) received a vehicle without either antigen or adjuvant. The incorporation of TLR agonists resulted in enhanced vaccine or therapeutic efficacy, as reported in 90% (46/51) of the studies (Table 5). Three studies (6%, 3/51) of the studies quantified the efficacy of vaccines that utilized TLR agonists either as adjuvants or therapeutic agents, including 57, 70, and 100% (35–37). Forty-one percent (21/51) of the studies reviewed investigated the utilization of multiple TLR agonists either in combination or as individual adjuvants. Among these, 43% (9/21) explored the synergistic effects of TLR agonists observing enhanced humoral and/or cellular immune responses with combined TLR agonists compared to singular TLR agonist application (Tables 5, 6). In summary, specific beneficial outcomes of using TLR agonists included reduced viremia, increased survival rate of animals, enhanced protection against diseases, and reduced clinical symptoms of the various infectious diseases evaluated (Figure 4).

**Table 5:** Efficacy outcomes of TLR agonists.

Outcomes measured	Total (%) *n* = 51
Application of TLR agonists	
Assess efficacy and immunogenicity	47 (92%)
Assess safety and immunogenicity	4 (8%)
Enhanced humoral response	
Yes	41 (80%)
No	10 (20%)
Enhanced cellular response	
Yes	41 (80%)
No	10 (20%)
Enhanced vaccine protective efficacy	
Yes	46 (90%)
No	5 (10%)

**Table 6:** Studies that evaluated the use of more than one toll-like receptor agonist.

TLR No. 1	TLR No. 2	TLR No. 3	TLR No. 4	Synergy assessment	Animal model	Disease model	Authors
TLR 1/2	TLR 9	TLR 7/8	N/A	Yes	Swine	Enzootic pneumonia	(70)
TLR 1/2	TLR 9	TLR 7/8	N/A	Yes	Swine	Porcine respiratory syndrome	(38)
TLR 4	TLR 21	TLR 3	N/A	No	Chicken	Mareks disease	(92)
TLR 21	TLR 3	TLR 2/4	N/A	No	Chicken	Avian influenza	(108)
TLR 1/2	TLR 7/8	TLR 9	N/A	No	Swine	Porcine respiratory syndrome	(78)
TLR 1/2	TLR 7/8	TLR 9	N/A	No	Swine	Porcine respiratory syndrome	(79)
TLR 1/2	TLR 7/8	TLR 9	N/A	No	Swine	Porcine respiratory syndrome	(77)
TLR 2	TLR 7	N/A	N/A	Yes	Swine	Porcine respiratory syndrome	(99)
TLR 7/8	TLR 5	N/A	N/A	No	Non-human primates	Influenza	(81)
TLR 3	TLR 4	N/A	N/A	No	Non-human primates	HIV	(88)
TLR 2	TLR 4	TLR 7	TLR 21	No	Chicken	Avian influenza	(65)
TLR 7/8	TLR 9	N/A	N/A	No	Non-human primates	Schistosomiasis	(63)
TLR 3	TLR 7/8	N/A	N/A	Yes	Non-human primates	Dengue viral infection	(93)
TLR 2	TLR 4	N/A	N/A	No	Swine	Porcine respiratory syndrome	(91)
TLR 2	TLR 5	N/A	N/A	No	Chicken	Avian influenza	(105)
TLR 3	TLR 4	TLR 21	N/A	Yes	Chicken	Avian influenza	(74)
TLR 4	TLR 7	N/A	N/A	Yes	Non-human primates	Simian Immunodeficiency	(87)
TLR 3	TLR 7/8	TLR 7	N/A	Yes	Chicken	Avian influenza	(83)
TLR 2	TLR 3	N/A	N/A	Yes	Chicken	Infectious bursal disease	(89)
TLR 9	TLR 7/8	N/A	N/A	No	Non-human primates	Simian Immunodeficiency	(67)
TLR 4	TLR 7/8	N/A	N/A	Yes	Non-human primates	Simian Immunodeficiency	(84)

### 3.5 Description of safety outcomes

The safety outcomes in the studies we reviewed were qualitatively assessed. Eight percent (4/51) of the studies evaluated the adverse effects of the TLR agonists as vaccine adjuvants. One of the studies, reported adverse effects, including fever, chronic inflammation, and granuloma in the use of TLR1/2, TLR9, and TLR8 agonists combination administered intramuscularly in swine (38) (Table 7).

**Table 7:** Adverse effects of TLR agonists.

TLR agonist	TLR receptor	Injection route	Adverse effects	Disease	Animal models	References
Pam3Cys, CpG ODN, Resiquimod	TLR1/2, TLR9, TLR8	Intramuscular	Fever, chronic inflammation, granuloma	*Mycoplasma hyopneumoniae*	Swine	(38)
3 M-052-SE	TLR7/8	Intramuscular	None	SARS-CoV-2	Rhesus Monkey	(80)
R848, Flagellin	TLR7/8, TLR5	Intramuscular	None	Influenza	Rhesus Monkey	(81)
GLA-SE	TLR 4	Intramuscular	None	Malaria	Rhesus Monkey	(86)

### 3.6 Quality of the studies

**Figure 5:**
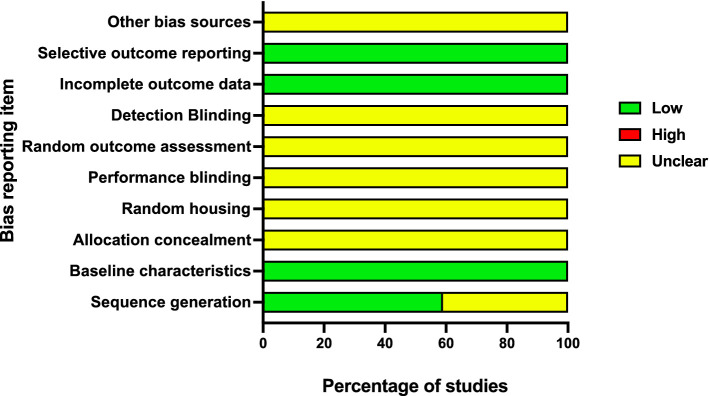
Summary of risk of bias score from all the studies in each domain according to SYRCLE protocol.

In approximately 59% (30/51) of the studies, randomization was reported, while 41% (21/51) of studies did not. According to the Systematic Review Centre for Laboratory Animal Experimentation (SYRCLE) risk of bias assessment, all the studies showed a low risk of bias from baseline characteristics, incomplete outcome data, and selective outcome (Figure 5). All the studies failed to report on allocation concealment, random housing, performance blinding, and detection binding, with 60% (31/51) reporting on sequence generation (Figure 5). All the studies clearly stated objectives and methodology, including types of data collected and the location of the study. Approximately 98% of the studies reported the sample size and the study groups. However, none of the studies indicated a statistical sample size calculation method.

## 4 Discussion

The review assessed the efficacy and safety of TLR agonists as therapeutic agents and vaccine adjuvants for infectious diseases. This study covers 51 studies. Our review shows TLR agonists are used more as vaccine adjuvants than therapeutic agents against infectious diseases in animals. As adjuvants, most TLR agonists effectively enhance immune response. They increased the efficacy of vaccines against infectious diseases, including viral, bacterial, protozoal, and parasitic infections, with viral infections attributing to 70% of the diseases assessed. A combination of TLR agonists resulted in synergistic effects with robust humoral and cellular immune responses that enhanced vaccine efficacy. Most of the studies did not evaluate the safety profile of the TLR agonists. Only 8% of the studies assessed adverse effects of specific TLR agonists. Adverse effects, including fever, chronic inflammation, and granuloma, were reported using a combination of TLR1/2, TLR9, and TLR8 agonists in swine.

TLRs play a crucial role in initiating innate immune responses and linkage to adaptive immune responses, rendering them promising targets for enhancing the efficacy of animal vaccines and therapeutics (18). The ability of TLRs to detect and respond to pathogens has highlighted their potential role as therapeutic agents in both infectious and noninfectious diseases. Targeting key processes in innate immunity could be explored for the prevention of infectious diseases in animals (39). By enhancing the host’s immune system, TLR agonists can mitigate the likelihood of infections. With the increasing global concern over antimicrobial resistance, there is a surge in identifying alternative therapeutics, and TLR agonists have been gaining popularity in this context over the years (1). In veterinary medicine, the growing risk of antimicrobial resistance necessitates the exploration of such alternatives. The strategic use of TLR agonists could potentially offer a means to combat infections while minimizing the reliance on traditional antibiotics, thus contributing to the global effort to manage and reduce antimicrobial resistance.

Recent developments in vaccinology have shown a surge in the utilization of non-pathogenic alternatives to traditional vaccines, including subunit vaccines, DNA vaccines, and mRNA vaccines. While these alternatives have gained demonstrated immunogenicity in murine studies, their efficacy in large animals remains limited. Consequently, there is a need to incorporate potent adjuvants to boost their immunogenicity (40, 41). Adjuvants act as immunostimulatory agents, and some act as vaccine delivery carriers, impacting the efficacy and safety of vaccine antigens (42). In the current review, TLR 9 agonists emerged as a popular TLR agonist for the infectious diseases reviewed. Other studies have reported high adjuvanticity in CpG ODN, a TLR 9 agonist, for diseases such as malaria, hepatitis B, HIV, anthrax, and COVID-19 (43). The wide application of TLR9 in this review indicates that it is effective in enhancing targeted immune responses in animals either as vaccine adjuvants or therapeutic agents against infectious diseases. This finding is consistent with other studies that have demonstrated the efficiency and effectiveness of TLR 9 and TLR 7 to clear viral pathogens (44, 45).

The synergistic effects of TLR agonists can be observed when they are used in combination with other adjuvants or therapeutic agents. For example, the combination of TLR3 and TLR9 agonists has been shown to significantly reduce clinical signs of canine herpesvirus infection (46). Similarly, the co-administration of vaccines with TLR agonists in combination with other adjuvants such as saponins has been shown to enhance protective immunity in livestock against bacterial and viral pathogens (47). Covalently linked TLR agonists confined in a particle format have also been shown to increase immune stimulation (48). These combinations leverage different aspects of the immune response, resulting in a more comprehensive defense against infectious agents. In the review, it was observed that combining TLR agonists resulted in a stronger immune response compared to using a single TLR agonist. Importantly, TLR agonists associated with T-cell responses are particularly desirable for therapeutics and vaccine efficacy against many infectious diseases in animals. TLR2, TLR3, TLR7, and TLR9 agonists have been shown to elicit CD8+ T cell responses and confer protective immunity against livestock disease pathogens, including Theileria spp., babesia spp., toxoplasma spp., Trypanosoma spp., and leishmania spp. (49).

Activation of TLRs by their ligands leads to a signaling cascade specific to the type of ligand, interacting receptors, and the adaptor molecules engaged in the signaling (50). However, pro-inflammatory cytokines and chemokines targeting pathogen clearance can result in excessive inflammatory reactions that can be harmful to the host (51). Furthermore, other studies have highlighted adverse effects in human trials related to formulations of TLR4, TLR 5, and TLR 9 agonists (52, 53). Generally, TLR agonists have been documented to have good safety profiles in cancer therapeutics in human trials. However, this review demonstrates the deficiency in establishing the safety profile of TLR agonists in animals *in vivo*, despite TLR agonists having a broad spectrum of pathogen-derived compounds and the complexity of species immunopathological responses, which may influence the side effects of TLR adjuvants (54, 55). In this review, non-human primates comprise the largest proportion of experimental animals, surpassing chickens, cattle, and swine. The popularity of non-human primates encountered in this review shapes the scope of infectious diseases investigated. Consequently, in this review, there was a significant scarcity of studies involving large animals such as cattle and pigs, leading to a bias in the infectious diseases covered in this review. Additional studies are essential to ascertain the safety profiles of TLR agonists in animals, as this factor profoundly impacts the efficacy outcomes of TLR agonists and, consequently, the efficacy of associated vaccines and therapeutics. Furthermore, the safety and efficacy of TLR agonists are affected by the route of administration, which is informed by their therapeutic purpose (56–58). In this review, the intramuscular and subcutaneous routes were the most common routes for TLR agonist administration. Local administration, such as the subcutaneous route, is more convenient than the systemic, intravenous route, and it has been shown to yield greater bioavailability than the oral route (56, 59, 60).

The application of TLR agonists shows great potential as prophylactic and therapeutic agents, as well as adjuvants, in veterinary research and medicine. This review demonstrates their effectiveness as potent adjuvants that can be incorporated into veterinary vaccines for infectious diseases to enhance efficacy and durability. Although the application of TLR agonists as therapeutics is currently limited, the reviewed studies highlight their potential as stand-alone therapeutics to boost immune responses in animals already infected with pathogens. The growing issue of antimicrobial resistance further emphasizes the need to develop alternative strategies, such as TLR agonists, to manage infectious diseases in animals.

The methodological limitations identified in this review highlight the ongoing challenges in the quality of methodology reporting in animal experiments, generally indicating potential reporting bias. Some studies did not mention randomization, which could introduce selection bias and confounding factors into the experiments (34, 57). Additionally, the failure to report allocation concealment, random housing, blinding, and detection blinding in all studies suggests potential selection bias and performance bias, which could compromise the validity and reliability of the findings (34). The absence of techniques for sampling and determination of sample sizes could lead to selection bias and affect the statistical power of the study, potentially resulting in inconclusive outcomes and ethical issues related to unnecessarily large or too small sample sizes (61). Addressing these methodological shortcomings in future animal research is essential to enhance the validity of research outcomes.

Moreover, this systematic review is subject to certain limitations that should be considered when interpreting its findings. The safety profile of TLR agonists presented in this study is not exhaustive. Given the rapid rise in the use of TLR agonists in veterinary medicine, evaluating and documenting any side effects associated with their use is crucial, no matter how mild they may seem. Murine-related studies, which comprise the majority of animal experimental studies, were excluded due to differences in physiological and pathological systems between large animals and murine models. These differences can significantly impact disease development and impede translational medicine efforts (62).

Future research should focus on assessing the safety profile of TLR agonists in large animals individually, rather than as part of a vaccine. Dose titration studies should be conducted to determine the appropriate dosages of TLR agonists for various animals, particularly when used as therapeutic agents. This could lead to improvements in their formulation and delivery methods, thereby maximizing their efficacy. Understanding the specific interactions between different TLR agonists and their safety profiles could enhance their therapeutic and adjuvant properties, targeting both synergy and the therapeutic potential of their antagonistic effects against specific infectious diseases in animals. Additionally, futute studies should incorporate a wider variety of animal models and large-scale field trials. These are necessary to validate the effectiveness of TLR agonists across diverse animal populations and environmental conditions.

## 5 Conclusion

Toll-like receptor agonists are efficacious immune response enhancers and thus have great potential in animal health interventions. Their synergistic effects when combining two or more TLR agonists or with other immune-modulating agents offer a powerful approach to improving disease outcomes both as vaccines and therapeutic agents. With the rising global concerns over antimicrobial resistance (AMR), the application of TLR agonists as alternative therapeutics could be revolutionary in veterinary medicine. Apart from mitigating AMR, these agents have the potential to be not only more cost-effective but also more efficient, as therapeutics like antibiotics are dosage-dependent on weight.

More effort should be devoted to collecting comprehensive data on the safety profile of TLR agonists. Understanding these safety profiles is crucial for identifying appropriate candidates for use across different animal species, considering variability in immunopathological responses, and informing the selection of safe and efficacious adjuvants.

## Data Availability

The original contributions presented in the study are included in the article/Supplementary material, further inquiries can be directed to the corresponding author.
